# The AKT Inhibitor MK-2206 is Cytotoxic in Hepatocarcinoma Cells Displaying Hyperphosphorylated AKT-1 and Synergizes with Conventional Chemotherapy

**DOI:** 10.18632/oncotarget.1236

**Published:** 2013-08-24

**Authors:** Carolina Simioni, Alberto M. Martelli, Alice Cani, Rengul Cetin-Atalay, James A. McCubrey, Silvano Capitani, Luca M. Neri

**Affiliations:** ^1^ Department of Morphology, Surgery and Experimental Medicine, University of Ferrara, Ferrara, Italy; ^2^ Department of Biomedical and Neuromotor Sciences, University of Bologna, Bologna, Italy; ^3^ Institute of Molecular Genetics, National Research Council, Pavia, Italy; ^4^ Muscoloskeletal Cell Biology Laboratory, IOR, Bologna, Italy; ^5^ Department of Molecular Biology and Genetics, Bilkent University, Ankara, Turkey; ^6^ Department of Microbiology & Immunology, Brody School of Medicine, East Carolina University, Greenville, NC, USA

**Keywords:** Hepatocellular carcinoma, MK-2206, Akt-1, targeted therapy, apoptosis, autophagy

## Abstract

Hepatocellular carcinoma (HCC) is one of the most common potentially lethal human malignancies worldwide. Advanced or recurrent HCC is frequently resistant to conventional chemotherapeutic agents and radiation. Therefore, targeted agents with tolerable toxicity are mandatory to improve HCC therapy and prognosis. In this neoplasia, the PI3K/Akt signaling network has been frequently shown to be aberrantly up-regulated. To evaluate whether Akt could represent a target for treatment of HCC, we studied the effects of the allosteric Akt inhibitor, MK-2206, on a panel of HCC cell lines characterized by different levels of Akt-1 activation. The inhibitor decreased cell viability and induced cell cycle arrest in the G_0_/G_1_ phase of the cell cycle, with a higher efficacy in cells with hyperphosphorylated Akt-1. Moreover, MK-2206 induced apoptosis, as documented by Annexin V labeling, and also caused autophagy, as evidenced by increased levels of the autophagy marker LC3A/B. Autophagy was shown to be a protective mechanism against MK-2206 cytotoxicity. MK-2206 down-regulated, in a concentration-dependent manner, the phosphorylation levels of Akt-1 synergizedand its downstream targets, GSK3 α/β and FOXO3A. MK-2206 synergized with doxorubicin, a chemotherapeutic drug widely used for HCC treatment. Our findings suggest that the use of Akt inhibitors, either alone or in combination with doxorubicin, may be considered as an attractive therapeutic regimen for the treatment of HCC.

## INTRODUCTION

Hepatocellular carcinoma (HCC) is one of the most deadly cancers worldwide with only few therapeutic options for patients with advanced disease, since it usually develops on the background of chronic liver disease and conventional anticancer therapies are not effective [1]. For example, the patient response rate to doxorubicin, the most widely used chemotherapeutic agent for HCC, is between 2% and 10% [2]. Therefore, major efforts are being made to develop rationally targeted therapies against altered signaling cascades that sustain HCC cell proliferation, survival, and drug-resistance. Sorafenib, a Raf kinase inhibitor, became the first drug to receive FDA approval for HCC, after being demonstrated to increase post-diagnosis mean survival of patients with advanced HCC and cirrhosis from approximately 8 to 11 months [3-5]. These results have triggered the search for other additional molecular targets to further improve HCC patient survival [6, 7].

The PI3K/Akt signaling pathway plays a central role in regulating cell proliferation, migration, survival and angiogenesis [3, 8]. Activation of phosphoinositide dependent kinase 1 (PDK1) and Akt by class IA PI3Ks (which includes PI3K p110α) is negatively regulated by PTEN, that converts phosphatidylinositol-(3,4,5)-trisphosphate [PtdIns(3,4,5)P3] to phosphatidylinositol-(4,5)-bisphosphate [PtdIns(4,5)P2] [9]. However, this signaling pathway is involved not only in physiological processes, but also in the development of cancers, including HCC [8, 10-12]. In HCC, deregulation of the PI3K/Akt pathway is the result of multiple molecular mechanisms, including activating mutations of PI3K p110α catalytic subunit, loss of expression of its negative regulator, the lipid phosphatase and tensin homolog deleted on chromosome ten (PTEN) or aberrant activation of receptor tyrosine kinases [13]. PTEN was demonstrated to be involved in HCC pathogenesis and in increased tumor grade and poor prognosis. [14, 15].

Phosphorylation of Akt at Ser473 was detected in up to 71% of HCC samples, and was associated with invasion, metastasis and vascularization [16]. The same authors, using a panel of HCC cell lines, demonstrated that Akt-1 is widely represented and is the most abundantly expressed Akt isoform. Activated Akt is known to inhibit apoptosis through its ability to phosphorylate several targets, including BAD, FoxO transcription factors, Raf-1 and caspase-9, which are critical for cell survival [17].

However, the clinical relevance of the PI3K/Akt pathway as an innovative target in HCC and its therapeutic potential remain to be further elucidated, in parallel with our growing knowledge of the role of signaling pathways and their alterations involved in HCC pathogenesis.

MK-2206 is a novel, orally active, allosteric Akt inhibitor which is being tested both in preclinical settings and clinical trials as an anticancer agent. It can synergistically enhance the antitumor effect of some conventional chemotherapeutic drugs and molecular targeted agents in lung cancer, ovarian cancer, breast cancer and acute leukemias [18, 19].

In this study, we analyzed the cytotoxic activity of MK-2206 in HCC cell lines displaying different levels of Akt-1 phosphorylation. We documented that MK-2206 was much more cytotoxic to cell lines (Mahlavu and SNU475) displaying higher levels of Akt-1 activation than to cell lines with lower levels of activated Akt-1 (PLC, SNU387). Treatments of HCC cells with MK-2206 caused cell cycle arrest in the G0/G1 phase of the cell cycle, induced apoptosis and autophagy. However, autophagy was a protective mechanisms against MK-2206 cytotoxicity. Moreover, MK-2206 potently synergized with doxorubicin in Mahlavu cells. These findings suggested that targeting Akt-1 with MK-2206, alone or in combination with conventional chemotherapy, may represent a new promising therapeutic approach in the treatment of HCC with hyperphosphorylated Akt-1.

## RESULTS

### Akt-1 phosphorylation levels in HCC cell lines are related to PTEN expression

**Figure 1:**
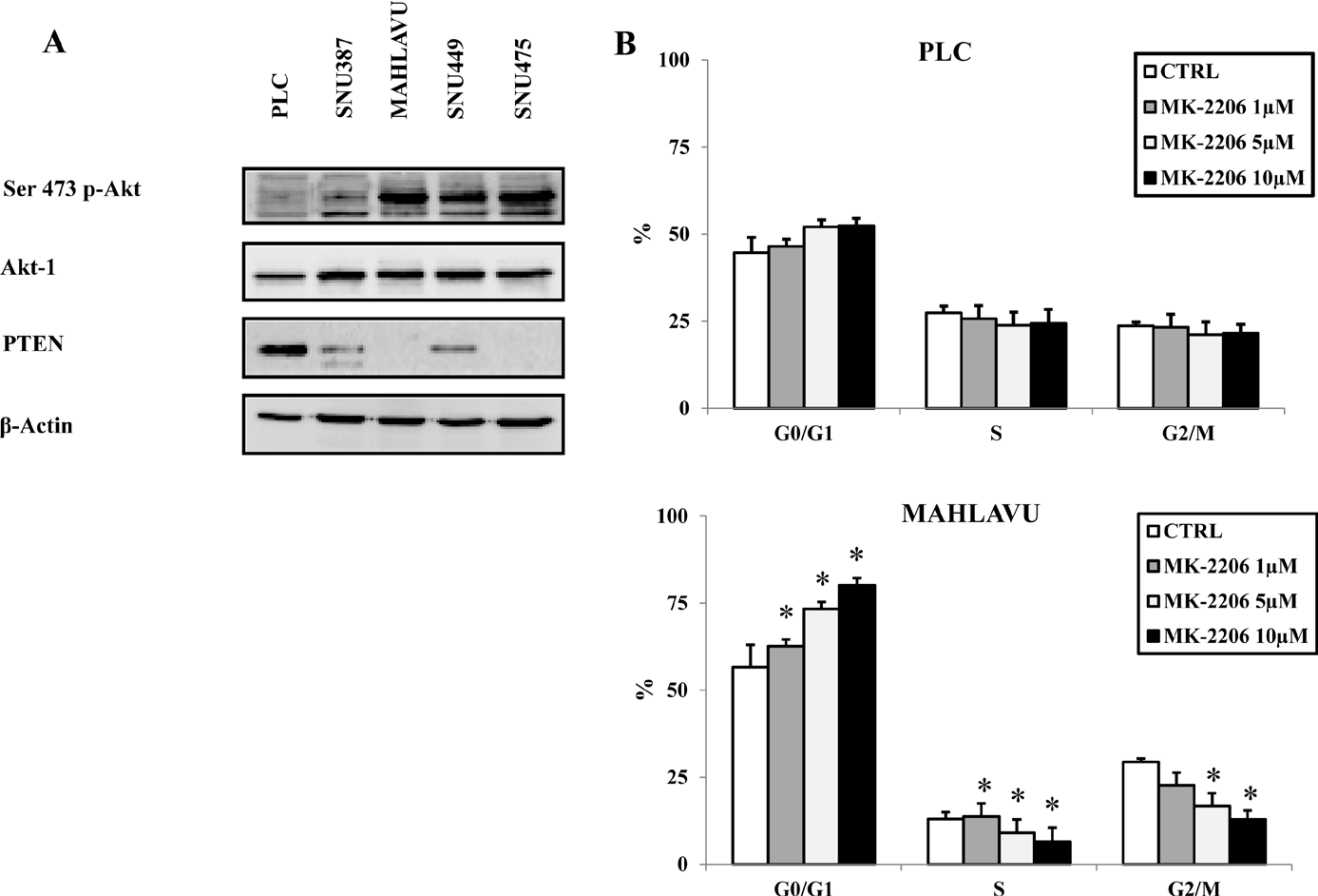
Different levels of Akt-1 phosphorylation are detected in HCC cell lines and correlate with cell cycle block induced by MK-2206 (A) Western blot analysis for Ser 473 p-Akt-1, total Akt-1 and PTEN in HCC cell lines. Fifty μg of protein was blotted to each lane. An antibody to β-actin documented equal lane loading. (B) Cell cycle in PLC and Mahlavu cells, treated with MK-2206, was analyzed by the Muse^™^ Cell Analyzer, according to the instrument protocol. The results are the mean ± s.d. of three different experiments. Asterisks indicate significant differences (p<0.05) in comparison with control (CTRL).

We first analyzed the basal expression of Akt-1 and its phosphorylation status on Ser473 on a panel of human HCC cell lines (PLC, SNU387, Mahlavu, SNU449 and SNU475 cells). Akt-1 total amount was similar in the five cell lines examined (Fig. 1A). On the contrary, the phosphorylation status of the protein, as documented by Western blot analysis with an antibody to Ser 473 p-Akt-1, showed relevant differences: in PLC cells a negligible phosphorylation level of Akt-1 was observable, in SNU387 cells only a slight Akt-1 phosphorylation was detectable, whereas a significant Akt-1 phosphorylation was detectable in Mahlavu, SNU449 and SNU475 cells. SNU387 cells in comparison with PLC cells showed a decrease of PTEN protein. A lower expression in SNU449 or loss of PTEN protein in Mahlavu and SNU475 cell lines, respectively, was associated with Akt-1 hyperphosphorylation. Therefore, our initial observations suggested that Mahlavu, SNU449 and SNU475 cells displayed hyperactivated Akt-1 when compared with PLC and SNU387 HCC cell lines.

### MK-2206 blocks in the G0/G1 phase of the cell cycle HCC cells with hyperphosphorylated Akt-1

Given the importance of the PI3K/Akt signaling pathway in the regulation of cell proliferation [20], the effects of MK-2206 on cell cycle progression of HCC cells were investigated. The analysis was carried out on PLC and Mahlavu cells, to compare the drug effect in an Akt-1 non-hyperphosphorylated cell line versus an Akt-1hyperphosphorylated one. Both cell lines were treated with increasing concentrations of MK-2206 for 24h, after which time the cells were harvested, fixed and stained with Propidium Iodide (PI) for the MuseTM Cell Analyzer. The analysis documented a not significant increase in the G0/ G1 phase of the cell cycle in PLC cells whereas a striking concentration-dependent increase in the G0/G1 phase of the cell cycle and a concomitant decrease in the S and G2/M phases were detected in Mahlavu cells (Fig. 1B).

### Real-time, dynamic monitoring of cell growth in HCC cells treated with MK-2206

**Figure 2:**
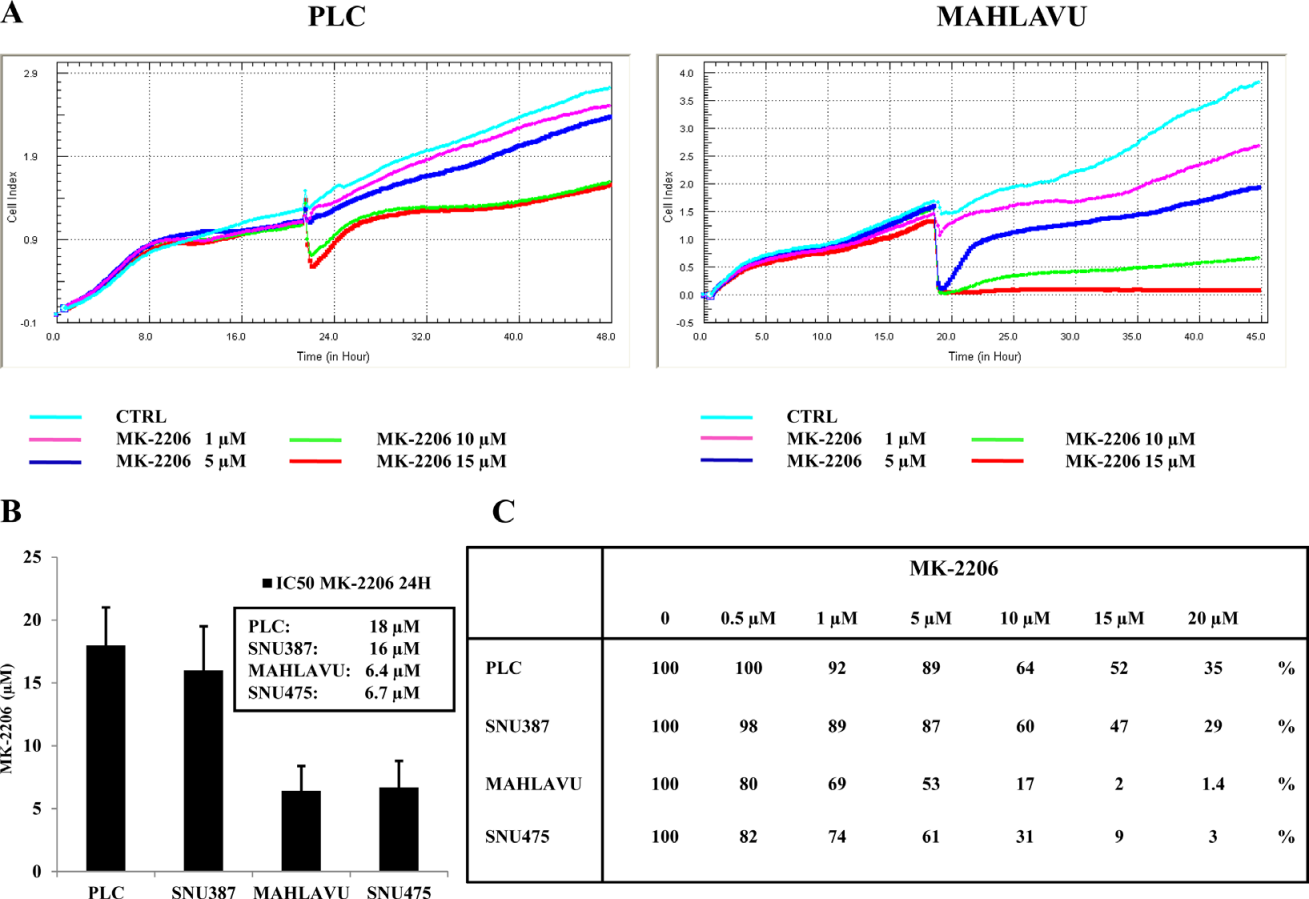
Dynamic monitoring of cell growth in HCC cells with the xCELLigence System (A) Concentration- and time- dependent cytotoxic effects of MK-2206 in PLC and Mahlavu cells after MK-2206 treatment, analyzed using the xCELLigence System. (B) IC_50_ values of MK-2206 at 24h of treatment in PLC, SNU387, Mahlavu and SNU475 cell lines. (C) CI values in PLC, SNU387, Mahlavu and SNU475 cell lines after 24h of treatment with MK-2206. One representative of three different experiments is shown.

To further analyze the activity of MK-2206 in HCC cells, we used a novel cell surveillance system to monitor the dynamic changes in cell growth based on the electrical impedance measurement technique (xCELLigence System). The xCELLigence System allowed us to study the effects of MK-2206 on HCC cells by a label-free and a real-time native approach. After the setting of the proliferation standards of PLC, SNU387, Mahlavu and SNU475 cells, we studied the cytotoxicity of the inhibitor at increasing concentrations. The plots demonstrated the concentration- and time-dependent cytotoxic effect of MK-2206 on PLC and Mahlavu cells (Fig. 2A) and the IC50 values of the drug at 24h of treatment was obtained by this technique: 18 μM for PLC, 16 μM for SNU387, 6.4 μM for Mahlavu and 6.7 μM for SNU475 cells, respectively (Fig. 2B).

We also analyzed the Cell Index after 24h of treatment with MK-2206 at different concentrations. As shown in Fig. 2C, Mahlavu and SNU475 cells were more sensitive to MK-2206 than PLC and SNU387 cells. It is noticeable a correlation between the lower concentration of drug needed to attain IC50 values and the levels of hyperphosphorylated Akt-1 in Mahlavu and SNU475 cells.

### MK-2206 induces both apoptosis and autophagy in HCC cells

**Figure 3:**
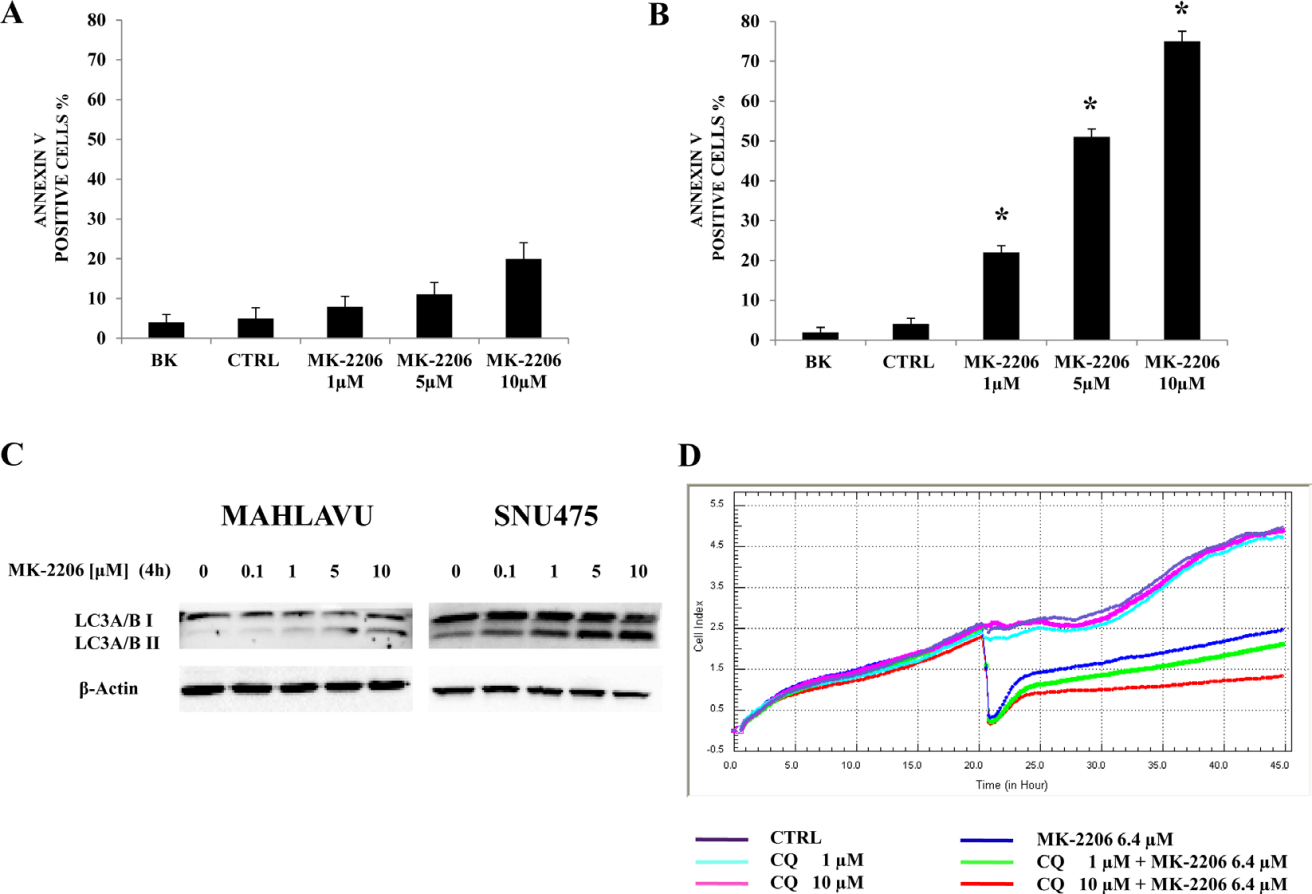
MK-2206 induces apoptosis and autophagy in HCC cell lines (A, B) Analysis of Annexin-V positive cells after MK-2206 treatment using the Muse^™^ Cell Analyzer in PLC and Mahlavu cells. The analysis was performed after 24h of treatment with increasing concentrations of MK-2206. The results are the mean ± s.d. of three different experiments. Asterisks indicate significant differences (p<0.05) in comparison with control (CTRL). BK represents the unstained samples. In both cell lines the apoptotic process was further increased when the drug was administered at the concentration of 10 μM. (C) Western blot analysis documenting increased expression of the fast-migrating (lipidated) form of LC3A/B in Mahlavu and SNU475 cell lines treated with MK-2206. An antibody to β-actin documented equal lane loading. (D) xCELLigence System analysis documenting the effects of chloroquine (CQ) on growth of Mahlavu cells treated with MK-2206. One representative of three different experiments is shown.

In order to establish whether decreased cell growth was related to apoptosis in HCC cell lines, we analyzed programmed cell death by Annexin-V/7-AAD-assay using the MuseTM Cell Analyzer. PLC and Mahlavu cells were treated with increasing concentrations of MK-2206 for 24h and then analyzed for Annexin-V labeling. MK-2206 induced concentration-dependent apoptosis in both cell lines. However, at 5 μM concentration of MK-2206, apoptosis was 11.7% in PLC cells, (Fig. 3A), whereas it reached 51.6 % in Mahlavu cells (Fig. 3B).

Recently, the inhibition of Akt has been shown to possess autophagy-inducing effects in addition to pro-apoptotic effect [21]. Induction of autophagy can promote either cell survival or cell death, depending on the cellular context and/or initiating stimulus [22].

To examine the effects of MK-2206 on autophagy in human HCC cell lines, we treated Mahlavu and SNU475 cell lines with the Akt inhibitor and then we assessed the conversion of LC3A/BI to LC3A/BII, as an autophagy marker [23-25]. Western Blot analysis documented, after 4h of treatment with MK-2206, an increase in the lipidated form of LC3A/B (LC3A/BII) in the two cell lines (Fig. 3C). To understand if autophagy was either a cell survival or a cell death mechanism, we inhibited this process using the lysosome inhibitor chloroquine [26] and then measured the Cell Index in Mahlavu cells. Chloroquine alone did not affect Mahlavu cell growth, even at the concentration of 10 μM. However, when Mahlavu cells were simultaneously treated with chloroquine and MK-2206, administered at the IC50 value, they became more sensitive than to MK-2206 alone, i.e. MK-2206 became more cytotoxic in the presence of 10 μM chloroquine (Fig. 3D).

### MK-2206 regulates PI3K/Akt signaling and induces MEK/ERK1/2 upregulation

**Figure 4:**
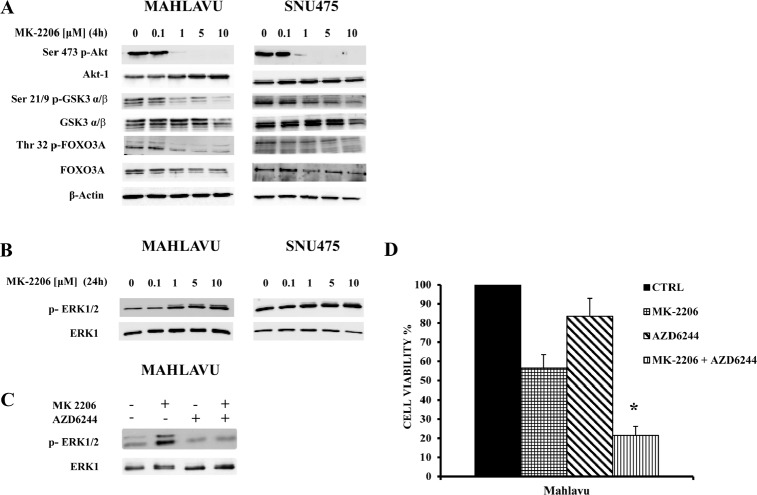
Effects of MK-2206 on the phosphorylation levels of key Akt substrates and ERK 1/2 (A) Western blot analysis for phosphorylated/total Akt-1, GSK3-α/β and FoxO3A in Mahlavu and SNU475 cells treated with increasing concentrations of MK-2206. 50 μg of protein was blotted to each lane. β-actin served as a loading control. (B) Western blot analysis for phosphorylated ERK1/2 and total ERK1 in Mahlavu and SNU475 cells treated with increasing concentrations of MK-2206. 50 μg of protein was blotted to each lane. (C) Western blot analysis for phosphorylated ERK 1/2 and total ERK1 in Mahlavu cells treated for 24 h with MK-2206 (5 μM) and/or AZD6244 (500 nM). (D) Cell viability analysis by MTT assays of Mahlavu cells treated for 24 h with MK-2206 (5 μM) and/or AZD6244 (500 nM). The results are the mean ± s.d. of three different experiments. The asterisk indicates significant differences (p<0.05) in comparison with control (CTRL) and single treatments.

In order to investigate the effects of MK-2206 on Akt-1 and its downstream targets, we analyzed the modulation of the PI3K/Akt pathway in Mahlavu and SNU475 cells. After a 4h incubation with MK-2206, a concentration-dependent decrease in p-Akt-1 levels was detected in all cell lines (Fig. 4A). The decrease was already detectable at 1 μM. In contrast, total Akt-1 levels were not affected by MK-2206. Akt inhibition had functional consequences on the phosphorylation of two well-established Akt substrates, GSK3-α/β and FoxO3A. Both proteins displayed dephosphorylation at amino acidic residues (Ser 21/9 for GSK3-α/β and Thr-32 for FoxO3A) that are targeted by Akt. The amounts of total GSK3-α/β and FoxO3A were unchanged.

Compensatory activation of parallel signaling through the MEK/ERK1/2 pathway in response to PI3K/ Akt inhibition, is an emerging theme in cancer cell signal transduction. Indeed, several recent reports have highlighted the importance of functional cross talks between the MEK/ERK1/2 and PI3K/Akt signaling networks, in response to individual pathway inhibitors [20, 27-31].

For this reason, we examined the status of p-ERK1/2 phosphorylation in both Mahlavu and SNU475 cell lines, after treatment for 24h with increasing concentrations of MK-2206. In both cell lines, we detected a concentration-dependent increase in the phosphorylation levels of p-ERK1/2, whereas the total protein amount remained unchanged (Fig. 4B).

To establish if this activation was dependent on MEK activity, we inhibited MEK in Mahlavu cells with the MEK-specific allosteric inhibitor, AZD6244 [32]. As shown in Fig. 4C, the drug completely prevented the MK-2206 induced up-regulation of p-ERK1/2.

Furthermore, we performed an MTT assay to determine if a combined treatment (MK-2206 plus AZD6244) would further decrease cell viability at 24h. When the two drugs were administered together, cell viability decreased significantly when compared with either drug alone (Fig. 4D).

### Down-regulation of Akt-1 reduces MK-2206 cytotoxicity in Mahlavu cells

**Figure 5:**
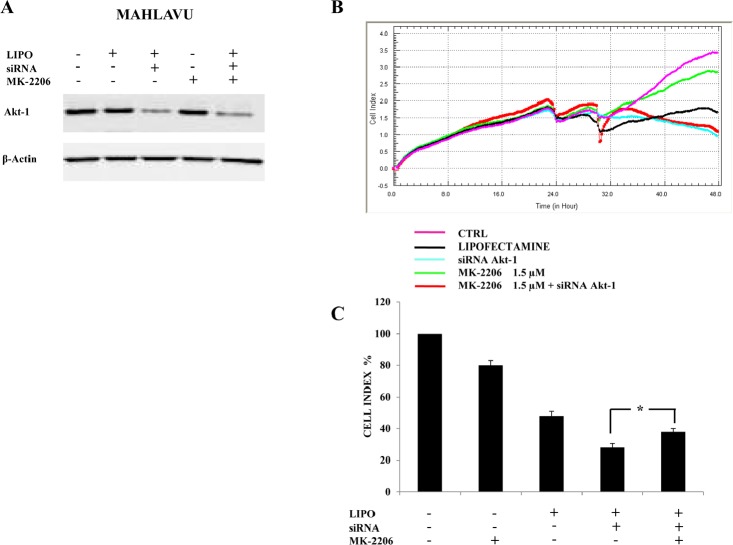
Down-regulation of Akt-1 reduces MK-2206 cytotoxicity in Mahlavu cells (A) Western blot analysis for Akt-1 in cells transfected for 24h with siRNA to Akt-1 and effects of MK-2206. LIPO: cells treated with Lipofectamine alone. β-actin served as a loading control. (B, C) xCELLigence System analysis documenting the effects of MK-2206 (18h of treatment) on cell growth, starting after 6h of transfection with siRNA to Akt-1. In (B) one representative of three different experiments is shown. In (C) the results are the mean ± s.d. of three different experiments. The asterisk indicate significant differences (p<0.05).

To further evaluate the inhibition of Akt-1 signaling as a major molecular target responsible for the effects of MK-2206 in HCC cells, we down-regulated protein expression of Akt-1 in Mahlavu cells by using siRNA. After 24h of transfection, we first examined by Western blotting the decrease of Akt-1 expression. As shown in Fig. 5A, Akt-1 siRNA significantly reduced the expression of Akt-1 protein. Down-regulation of Akt-1 was not further increased by administration of MK-2206.

Then, we studied cell growth with the xCELLigence System in both non-transfected and transfected cells. After 6h from transfection, MK-2206 was added in both non-transfected and transfected cells for an additional 18h. (Fig. 5B and C). Interestingly, the addition of MK-2206 to 6h transfected cells did not decrease their growth that resulted, on the contrary, higher than the siRNA treated sample. Therefore, the removal of MK-2206 target by transfection resulted in a lower drug cytotoxicity when compared with the drug-untreated transfected samples (Fig. 5B and C).

### MK-2206 synergizes with doxorubicin in Mahlavu cells

**Figure 6:**
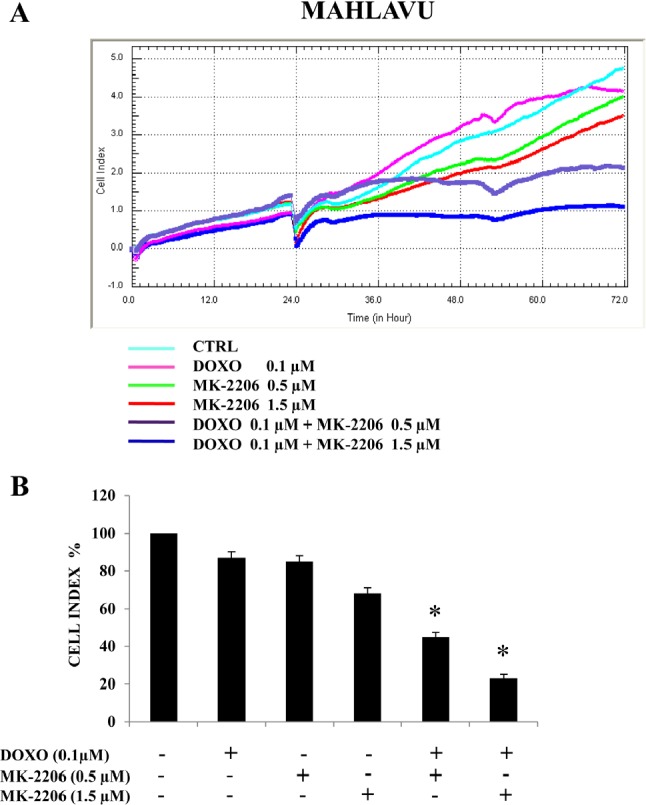
MK-2006 displays synergistic cytotoxic effects with doxorubicin in Mahlavu cells (A, B) xCELLigence System analysis showing the synergistic effects of a combined treatment consisting of doxorubicin (DOXO) at a single concentration (0.1 μM) and MK-2206 at two different concentrations (0.5 and 1.5 μM). The CI was monitored after 48h of treatment. In (A) one representative of three different experiments is shown, while in (B) the results are the mean ± s.d. of three different experiments. Asterisks indicate significant differences (p<0.05) in comparison with single treatments.

We then examined whether MK-2206 could synergize with the anthracycline antibiotic doxorubicin, since this drug is frequently included in different protocols to treat HCC. Mahlavu cells were treated with doxorubicin at a single concentration (0.1 μM) and with MK-2206 at two different concentrations (0.5 and 1.5 μM) and the synergistic effects of the combination were monitored after 48h or 72h of treatment using the xCELLigence System and by analyzing the Cell Index (Fig.6A and B). We could observe a synergistic effect of the combined drug treatment in suppressing cell proliferation and viability, more evident at the higher (1.5 μM) concentration of MK-2206 (Fig.6A and B).

## DISCUSSION

HCC is the third most common cause of cancer-related death worldwide [33]. Although patients with early-stage disease have a good prognosis, there has been no effective therapy available for those with advanced disease. Thus, treatment of HCC remains an urgent health concern [34].

Activation of the PI3K/Akt signaling pathway through mutations and/or inactivation of key pathway components occurs in various malignancies, including HCC [35]. Several mechanisms may be responsible for the activation of PI3K/Akt in HCC cells. The high frequency of the PI3K p110α gene (PIK3CA) mutations and/or its up-regulation in patients with a shorter survival is responsible for the Akt hyperactivation found in HCC with poor prognosis [20]. Moreover, impaired expression of PTEN could be involved in the regulation of PI3K/Akt activity. Indeed, activation of Akt signaling and a reduced expression of PTEN has been reported in 40–60% of human HCC [14]. Recent studies have demonstrated that PTEN expression plays a critical role in HCC progression and patient survival. Patients with a high PTEN expression had a significantly better overall survival than patients with a lower expression [36].

On the other hand, activated ERK1/2 and its downstream effectors were strongly upregulated in the miscroscopic, residual lesions in the livers of Akt/Ras mice [13], thus indicating the potential existence of a functional crosstalk between PI3K/Akt and Ras/Raf/MEK/ERK1/2 pathways along hepatocarcinogenesis, whose inhibition might be highly beneficial for the treatment of HCC patients.

Although some preclinical studies have demonstrated that PI3K/Akt inhibitors such as perifosine, LY294002 and wortmannin displayed anti-HCC activity, no studies have been conducted so far at the clinical level.

In this study, we aimed to investigate the potential therapeutic activity of the novel oral allosteric Akt inhibitor, MK-2206. In a recent investigation, the combination of the multikinase inhibitor Sorafenib and MK-2206 overcame the resistance of HCC cells to Sorafenib at clinically achievable concentrations, suggesting the potential use of this treatment in HCC patients [37]. Here, we demonstrated that MK-2206, as a single agent, decreased the growth of HCC cell lines in a concentration-dependent manner, blocked the cells in G0/G1 phase of the cell cycle and induced apoptosis. In particular, the activity of the drug on cell proliferation was analyzed through a novel, innovative technology referred to as xCELLigence System, where proliferation of cells, as a first step, and the cytotoxicity of the drug as a second step, were monitored in real-time through gold microelectrode arrays on a glass substrate in the bottom of 96 E-Plates, specific for the system. The cytotoxic activity of MK-2206 was more potent in cell lines with higher levels of p-Akt1 (Mahlavu, SNU475) than in cells with lower p-Akt1 levels (PLC, SNU387). This finding demonstrated the selectivity of the inhibitor.

An interesting observation is that MK-2206 also induced autophagy in HCC cells, as documented by the increased expression of lipidated LC3A/B (LC3A/B-II) in a concentration-dependent manner. The correlation between autophagy and tumorigenesis has been explored extensively, but whether autophagy acts as a pro-tumorigenic or anti-tumor player in tumor development and cancer therapy is still unclear [38, 39].

The process of autophagy is characterized by the formation of the autophagosome, a double membrane structure that sequesters the target organelles/proteins and then fuses with endo/lysosomes, where the content itself and the autophagosome major component, LC3A/B, are degraded [40, 41]. Autophagy has been demonstrated to be required for continued cell growth in pancreatic cancers [42]. Accumulating evidence demonstrated also that suppression of the proteins involved in autophagy, such as Beclin-1 and Atg-5, could cause acceleration of tumorigenesis [38]. We documented that chloroquine, an autophagy inhibitor, sensitized Mahlavu cells to the cytotoxic effects of MK-2206. This finding suggests that in HCC cells autophagy could have a tumor protecting role when neoplastic cells are treated with Akt inhibitors. In this connection, it is worth remembering here that it has previously been reported that MK-2206 induces autophagy in human glioma cells and this protected tumor cells against apoptosis [18]. Therefore, the inclusion of autophagy inhibitors could be considered in future HCC therapeutic protocols based on Akt inhibitors. Indeed, a number of clinical trials are now revealing the promising role of chloroquine as a novel antitumor drug [43].

In Mahlavu and SNU475 cell lines, MK-2206 dephosphorylated Akt-1 on Ser 473 and its downstream targets, GSK3-α/β and FoxO3A. However, MK-2206 increased the phosphorylation levels of ERK 1/2, through a MEK-dependent mechanism, as the hyperphosphorylation was blocked by the MEK inhibitor, AZD6244. ERK 1/2 hyperphosphorylation acted as a protective mechanism against MK-2206 cytotoxicity, as the MK-2206/AZD6244 combined treatment was more cytotoxic to Mahlavu cells than either treatment alone.

To further delineate the inhibition of Akt signaling as a major molecular target responsible for the effects of MK-2206 in HCC cells, we down-regulated protein expression of Akt-1 in Mahlavu cells by siRNA transfection. The viability of transfected cells with Akt-1 down-regulation, was higher than control samples after MK-2206 treatment, implying that removal of the MK-2206 target by transfection could result in a lower drug cytotoxicity.

Conventional anticancer therapies for the treatment of HCC are not effective [1]. Considering the very low response rate to doxorubicin, perhaps the most widely used traditional chemotherapeutic drug used in HCC patients [2], it appeared of interest to explore the efficacy of the combination of chemotherapy with other selective agents targeted to specific signal transduction effectors. We demonstrated in this work that MK-2206 was able to synergize with doxorubicin. Analysis of the results demonstrated in Mahlavu cells a synergistic effect of the combined treatment in suppressing cell proliferation.

In conclusion, our findings strongly suggest that MK-2206, either alone or combined with traditional chemotherapeutic drugs, could be a valuable compound for treating HCC patients displaying activation of PI3K/Akt signaling and who are still facing a very poor prognosis.

## MATERIALS AND METHODS

### Materials

Dulbecco's modified Eagle's medium (DMEM), RPMI-1640 medium, fetal bovin serum (FBS), nonessential amino acids (NEA), penicillin and streptomycin were from Lonza (Lonza Milano SRL, Milan, Italy). Opti-MEM® Reduced Serum Medium used for transfection was from Life Technologies (Invitrogen, Milan, Italy). For cell viability determination, Cell Proliferation Kit I (MTT) was purchased from Roche Applied Science (Basel, Switzerland). MK-2206 and AZD6244 were provided by Selleck Chemicals (Houston, TX, USA). Stock solutions of MK-2206 and AZD6244 were prepared in DMSO. Doxorubicin was obtained from Sigma-Aldrich (St. Louis, MO, USA). Antibody to total Akt-1 was from Santa Cruz Biotechnology (Santa Cruz, CA, USA) while all the other antibodies were from Cell Signaling Technology (Danvers, MA, USA), including an antibody specific for Ser473 p-Akt-1. siRNA to Akt-1 was from Santa Cruz Biotechnology.

### Cell culture and Western blot analysis

The HCC cell lines Mahlavu and PLC were maintained in DMEM medium supplemented with 10% FBS, 2 mM L-Glutamine, 0.1 mM NEA, 100 U/ml penicillin and 100 μg/ml streptomycin. SNU387, SNU449 and SNU475 cell lines were maintained in RPMI-1640 medium supplemented with 10% FBS, 2 mM L-Glutamine, 100 U/ml penicillin and 100 μg/ml streptomycin. All cells were cultured in a 37°C humidified incubator and an atmosphere of 5% CO2 in air. Western Blot analysis was performed by standard methods as described elsewhere [44-46].

### Real-time cell growth surveillance by cell electronic sensing (xCELLigence System)

Proliferation was monitored in real-time cell electronic sensing (xCELLigence System, Roche Applied Science) through gold microelectrode arrays on a glass substrate in the bottom of 96 E-Plates, specific for the system. HCC cells (2,500-20,000 cells/well in 100 μl, depending on the cell growth) were seeded into 96 E-Plates (Roche Applied Science) containing 100 μl medium/well. After 24h, 100 μl medium was discarded and replaced with 100 μl of fresh medium for each well. MK-2206 was added at the indicated concentrations to 200 μl of medium. In the xCELLigence System the changes in cell number are detected as modifications in the measurement of electrical impedance and are represented as Cell Index (CI). Since the number of cells that have been seeded is known, the CI is related to the quantitative measurement of the electrical impedance present in the well and it displays in plots the changes of cells that adhere to the conducting metals on the bottom of the wells. Therefore, CI values increase or decrease in parallel with cell growth due to the insulating properties of the cell membrane attached to the bottom of the well. Untreated cells were used to establish a reference baseline; results were baseline adjusted and expressed as CI normalized to the time point of compound administration. CI was used to determine also the IC50 values of MK-2206.

### Cell viability analysis

MTT (3-[4,5-Dimethylthythiazol-2-yl]-2,5-Diphenyltetrazolium Bromide) assays were performed to assess the sensitivity of cells to drugs, as previously described [47-49].

### Cell cycle and apoptosis analysis

Cell cycle analysis was performed using the MuseTM Cell Analyzer (Merck Millipore, Milan, Italy). In brief, after 24h of treatment, cells were harvested, centrifugedat 300 × g for 5 min and washed once with 1X PBS. After fixing them with 70% ethanol for at least 3h at −20°C, cells were centrifuged at 300 × g for 5 min, washed once with 1X PBS and then 200 μl of Muse TM Cell Cycle reagent was added to each tube with an incubation of 30 min at room temperature in the dark. Samples were then analyzed according to the manufacturer's instructions.

Moreover, analysis of apoptosis in PLC and Mahlavu cells was performed by Annexin-V/7-AAD-Assay using the MuseTM Cell Analyzer. In brief, cells treated with increasing concentrations of MK-2206 were harvested by trypsinisation after 4h and 8h of treatment, and a 100 μl cell suspension was labeled for 20 min in the dark with the same volume of the MuseTM Annexin-V & Dead Cell reagent (Merck Millipore). Subsequently, quantitative detection of Annexin-V/7-AAD positive cells was performed with the MuseTM Cell Analyzer.

### siRNA downregulation of Akt-1

Mahlavu cells were transfected in Opti-MEM® Reduced Serum Medium (Invitrogen) with 4μg siRNA Akt-1 in six-well plates using Lipofectamine 2000 (Invitrogen), according to the manufacturer's instructions. After 6h of transfection, Mahlavu cells were incubated for an additional 18h with MK-2206 for a total of 24h of transfection and then harvested for Western blotting analysis. The same experiment was performed using xCELLigence, where the cells where plated and transfected in 16 well xCELLigence plates.

### Combined drug effects analysis

The combination effect and a potential synergy between MK-2206 and doxorubicin were evaluated with the xCELLigence System. In brief, after 24h from seeding in E-Plate, Mahlavu cells were treated with different concentrations of MK-2206 and doxorubicin, and the combination effects were evaluated after 48h of treatment by monitoring the different values of CI.
